# Seasonal Changes in Plasma Levels of Sex Hormones in the Greater Rhea (*Rhea americana*), a South American Ratite with a Complex Mating System

**DOI:** 10.1371/journal.pone.0097334

**Published:** 2014-05-16

**Authors:** Diego J. Valdez, Marilina Vera Cortez, Natalia S. Della Costa, Alvina Lèche, Cristian Hansen, Joaquín L. Navarro, Mónica B. Martella

**Affiliations:** 1 Instituto de Diversidad y Ecología Animal (IDEA-CONICET), Centro de Zoología Aplicada, Universidad Nacional de Córdoba (UNC), Córdoba, Argentina; 2 Laboratorio de Análisis Clínicos Especializados (LACE), Córdoba, Argentina; Pennsylvania State University, United States of America

## Abstract

Seasonal rhythm in sex hormones has been extensively studied in birds, as well as its relationship with the type of mating system. The Greater Rhea (*Rhea americana*), a South American ratite species, reproduces seasonally and has a complex mating system: female-defense polygyny and sequential polyandry. The present study aimed at analyzing the endocrine basis of reproduction in this species and its relationship with its mating system. We used HPLC and electrochemiluminescence techniques to identify and measure plasma testosterone and estradiol levels. Annual oscillations in sex hormones, testosterone and estradiol, in adult males and females were observed. Lower levels of these hormones were exhibited during the non reproductive season (February to July), whereas their maximum values were reached in September for males and November-December for females. These fluctuations reflect the seasonal changes in gonadal function. By contrast, no significant sex hormones oscillations were observed in juvenile males and females (negative control of seasonal changes). Greater rheas maintain high testosterone and estradiol levels throughout the reproductive period. The high testosterone levels during incubation and chick rearing did not inhibit parental behavior in males, which appears not to conform to the “Challenge Hypothesis”. In females, the high estradiol levels throughout the reproductive season would be needed to sustain their long egg-laying period.

## Introduction

The occurrence of seasonal oscillations in both plasma and fecal levels of sex hormones (testosterone and estradiol) has been widely reported in birds. During the non-reproductive season sex hormone levels are low, becoming high in the reproductive season and leading to changes in courtship behavior in both males and females. This hormonal pattern has been observed in a long list of species with different mating systems [1], [2].

The seasonal pattern of testosterone in males has been related to mating systems for different species in the context of “The Challenge Hypothesis” [3], which postulates that high testosterone levels throughout the breeding season inhibit parental behavior in polygamous birds. This hormonal pattern allows polygamous males to copulate with all receptive females during the entire reproductive period. Monogamous species on the other hand exhibit high testosterone levels only during territorial establishment and mate selection, with low testosterone levels during incubation and parental care. The complex mating system of the Greater Rhea (*Rhea americana*), combining polygyny and sequential polyandry [4], [5], makes it an interesting avian model for the study of seasonal patterns of sex hormones and their relation to mating systems. This ratite reproduces seasonally from August to January and the breeding season commences with fights among the males, followed by courtship and copulation [6], [7]. Later, males build the nest scrape in the ground, incubate the eggs and take care of the chicks for months after the end of the breeding season [8], [9]. In addition, females move in non-cohesive groups (Martella et al. unpubl. data) and copulate and lay eggs in communal nests throughout the entire reproductive season [10], [11].

Although seasonal variations in sex hormones have been studied in other ratite species, such as the Ostrich, Emu and Kiwi [12]–[15], the endocrine basis underlying the mating system of the Greater Rhea remains unexplored, probably because of the difficulties involved in performing such studies in this species: on the one hand, the wild Greater Rhea populations are found in low densities [16], [17] and are currently considered to be near threatened [18]; and on the other, their successive capture for marking, blood sampling and subsequent behavior monitoring are extremely difficult to carry out in the wild. For these reasons, and given that the reproductive behavior of captive Greater Rhea males and females does not differ from that observed in the wild [6], [19], [20], we decided to carry out this study under controlled conditions of captivity as a first approach.

Based on the complex mating system of Greater Rheas, and under the assumption that rheas present seasonal changes in their plasma levels of sex hormones that reflect changes in gonadal function and reproductive behavior, we expect that testosterone and estradiol will start to increase at the beginning of the reproductive season, will remain high and then decline at the end of the reproductive season. By contrast, we suppose that male and female rhea juveniles (younger than one year old) will not show seasonal changes in sex steroids throughout the year due to their sexual immaturity.

The relationship between plasma testosterone levels and the reproductive and parental behavior in adult males will be discussed within the context of “The Challenge Hypothesis”.

## Materials and Methods

This study was carried out in strict accordance with the Guide for Ethical Research on Laboratory, Farm and Obtained from Nature Animals. It was approved by the ethics committee of the Consejo Nacional de Investigaciones Científicas y Técnicas (CONICET) (Resolution No. 1047 ANNEX II, 2005) before its implementation, as part of the author's postdoctoral fellowship project.

### Animals

We used 21 adult Greater Rheas (6 males and 15 females above 2 years of age) and 10 juvenile Greater Rheas (5 males and 5 females, all below 1 year old bred in captivity at an experimental farm in Córdoba Zoo, Argentina (31°25'31.79"S, 64°10'29.92"W).

Adult Greater Rheas were housed in three pens (600 m^2^ each) with natural soil floor. In each pen, 2 males and 5 females (resembling the sex ratio of reproductive groups observed in the wild) were provided with food (Vasquetto®) and water *ad libitum*. All adult birds were exposed to natural light and temperature conditions and had access to a 10-m^2^ roofed shelter.

Juvenile Greater Rheas (sexually immature individuals used as a negative control of seasonal hormone changes) were housed separately from adults in a 50 m^2^-pen with natural soil floor and were provided with processed chicken food (Vasquetto®) and water *ad libitum*. They were exposed to natural light and temperature conditions and had free access to a shelter with heat lamps. Before this study, all birds were sexed and identified following the method (DNA test) described by Rossi Fraire and Martella (2006) [21].

### Experimental design and blood sample collection

Adults and juveniles were sampled once a month by two zoo veterinarians during the non-reproductive season (April, May and July 2011 and February 2012) and the reproductive season (August 2011 to January 2012).

The animals were captured and immobilized by two experienced zoo assistants following experimental protocols that conform to international ethical standards [22].

Blood samples (3 mL in adults and 1.5 mL in juveniles) were obtained between 9:00 and 11:00 am and within 3 min of capture (to avoid an increase in glucocorticoid concentrations) from the right jugular vein using heparinized syringes [23], [24]. Samples were centrifuged (2500 G) for 15 min and the resulting plasma was collected and stored at −20°C until assayed.

### Behavioral sampling

In order to corroborate that the Greater Rheas in captivity exhibit the same behavior as the wild life, we filmed their reproductive behavior.

We focused mainly on reproductive behavior of males because that of females has no distinguishable characteristics, except for egg laying [25]. In the same week, but before blood sample collection (four samples during the non-reproductive season and six during the reproductive season), behavior was recorded according to Sales et al. (2000) [20]. Greater Rheas were video-recorded with a SAMSUNG smart camcorder equipped with a 52× of optical zoom. Videos were recorded during three time intervals were taken throughout the day (first: 10 to 12 am; second: 12 to 14 pm and third: 14 to 16 pm). Also, in each interval time three video recordings were made (10 min each) and observations were carried out on a particular pen and all birds were filmed simultaneously. Adult birds were observed individually, totaling 90 min (5400 seconds) of observation per month.

We focused on the following male reproductive behaviors: Courtship (wing display, vertical and lateral swinging movements of the neck), Incubation (male sitting on eggs) and Nest building (qualitative observation, male digging the ground with legs). The duration of each behavior was recorded in seconds.

### Hormone assay

We used high performance liquid chromatography (HPLC) technique to determine whether the antibodies included in the immunoassay kits were capable of tracking testosterone and 17β-estradiol in plasma of Greater Rhea.

We determined the testosterone and 17β-estradiol concentrations in plasma of Greater Rhea and in HPLC fractions, following procedures described in the commercial electrochemiluminescence immunoassay kits (*Elecsys Testosterone II and Elecsys Estradiol II* from *ROCHE*- it should be noted that kit does not require prior extraction). Both hormones were analyzed in *cobas* 6000 equipment, with a module for immunoassay *e 601* (HITACHI High Technology Corporation-ROCHE Diagnostic GmbH).

Manufacturer cross-reactivity in the testosterone kit was: Androstenedione (≤2.5%), Cortisol (≤0.01%), Estradiol (≤0.16%), Estrone (≤0.004%), Testosterone propionate (≤2.46%), 5-α-Androstene-3β, 17β-diol (≤2.11%), 5-α-dihydro-testosterone (≤0.86%). Manufacturer cross-reactivity in the 17β-estradiol kit was: Aldosterone (0.006%), Estriol (0.077%), Progesterone (0.009%), Estrone (0.0515%), 17β-estradiol-3,17-sulfate (0.411%), 17β-estradiol-17-sulfate (0.002%), 17-Hydroxyprogesterone (0.010%), Pregnenolone (0.008%).

To avoid any misinterpretation due to the transformation of the ln ng/mL data (since values lower than 1 become negative in logarithms), the testosterone concentration was expressed as ln nanograms of hormone per deciliter of plasma (ln ng/dL). 17β-estradiol was expressed as ln picograms of hormone per milliliter of plasma (ln pg/mL).

### High pressure liquid chromatography (HPLC): Testosterone and 17β-Estradiol

We used HPLC equipment with an autosampler and two detectors connected in series –DAD for estradiol and FD for testosterone– (AGILENT 1100) to identify testosterone and 17β-estradiol present in the plasma of male and female Greater Rheas, respectively. The elution positions of reference external standards of testosterone and 17β-estradiol (SIGMA) were determined by HPLC and then compared with the rhea samples. Both male and female plasma samples (September and December, respectively) were used to create a pool of presumably high testosterone and 17β-estradiol concentrations, respectively. Sex steroids from these pools (0.5 mL each) were extracted with a PHENOMENEX Strata C-18-E column for testosterone (≈70% recovery) and VARIAN Bond Elut C-18 column for 17β-estradiol (≈81% recovery). In both cases, steroids were eluted with 1 mL of ethyl acetate following the protocol of Koren et al. (2012) [26]. The solvent was evaporated with N_2_ (AIR LIQUID, Argentina) and the dry extracts were reconstituted in 100 µL of mobile phase.

For chromatographic runs of samples, 100 µL of plasma extract was injected onto a reversed-phase column (LiChrospher 100/RP-18/5 µm; 4.6×250 mm; Merk, Germany), with a mobile phase (1 mL/min) composed of 28% acetonitrile, 29% methanol and 43% water.

Furthermore, 31 HPLC fractions were also collected to determine the immunoreactive sex hormone component in Greater Rhea plasma in the validated immunoassay kits. All the HPLC fractions were dried and reconstituted in 500 µL steroid diluent provided in the kit. Finally, immunoreactivity profiles were obtained from the analysis of the collected eluates.

### Egg-laying period

The egg-laying period was assessed by counting eggs daily in the nests (in each pen) from September to January, to obtain the mean ± standard error of egg number laid in each month per pen.

### Statistical analyses

Statistical analyses were performed using the Infostat statistical software package [27]. The plasma testosterone and estradiol were ln-transformed for normality of residuals. We performed the following mixed models:

Two models to evaluate the effect of month (photoperiod) on testosterone levels in adult males (*Mod 1*) and juveniles (*Mod 2*), considering the dependent variable *hormone concentration*, the fixed factor *month* (10 months in adults and 6 months in juveniles), and the *identity* of the animal as the random factor.

A model to evaluate the effect of month and reproductive status of the animal (juvenile vs. adult) on testosterone levels, considering the dependent variable *hormone concentration*, fixed factors *month* (6 months in which data were available for both adults and juveniles), *reproductive status* and interaction *month-reproductive status*, and the *animal* as random factor (*Mod 3*).

Two models to evaluate the effect of month on estradiol levels in adult females (*Mod 4*) and juveniles (*Mod 5*), considering the dependent variable *hormone concentration*, the fixed factor *month* (10 months in adults and 9 months in juveniles) and the *animal* as random factor.

A model to evaluate the effect of month and reproductive status of the animal (juvenile vs. adult) on estradiol levels, considering the dependent variable *hormone concentration*, fixed factors *month* (9 months in which data were available for both adults and juveniles), *reproductive status* and interaction *month-reproductive status*, and the *animal* as random factor (*Mod 6*).

The contrast test was used for post-hoc analysis. Transformed values were expressed as the mean ± standard error (SEM) and the significance level was set at 0.05.

## Results

### High performance liquid chromatography (HPLC)

Chromatographic separation of pools of Greater Rhea plasma samples revealed that a peak co-eluted with the reference external standards of testosterone and 17β-estradiol, respectively. The immunoreactivity of the fractions obtained after HPLC show that the fraction corresponding to peaks of plasmatic testosterone and estradiol have maximum immunoreactivity values and the same retention time as external standards.

### Testosterone levels

**Figure 1:**
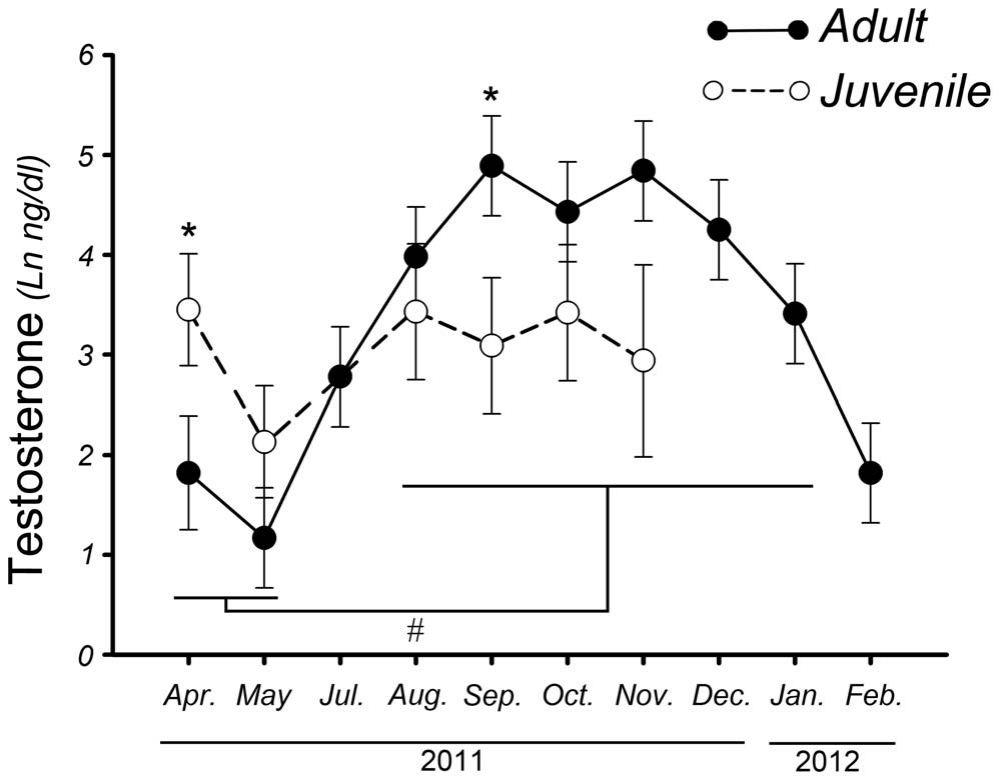
Annual changes in testosterone plasma levels of adult Greater Rhea males (solid line-filled circles) compared with juvenile rhea males (dashed line-empty circles), showing the differences between April and September (*). **#** Shows differences between April-May and August-January in Greater Rhea adult males. Results are expressed as mean ± standard error. Adult males n = 6, juvenile males (April n = 3) and (September n = 2); * and **#** denote *p*<0.01.

Adult plasma testosterone levels varied significantly among months (*F*_9, 43_ = 9.40, *p*<0.01, *Mod 1*, Fig. 1). We observed maximum testosterone levels from August to January, which were significantly higher (*p*<0.02) than those observed in February, April and May (non-reproductive period or baseline levels).

Juvenile plasma testosterone levels were not different among months (F_5, 4_ = 0.78, *p*>0.05, *Mod 2*, Fig. 1).

The observed changes in plasma testosterone levels among months showed an interaction with the breeding status (F_5, 27_ = 3.98, *p* = 0.01, *Mod 3*, Fig. 1). The testosterone levels in adults were different (*p*<0.05) than that of juveniles in April and September.

The coefficients of variation for nontransformed testosterone concentration (September, pool n = 6) was 3.6% (intra-assay) and 0.42% (inter-assay).

### Reproductive behaviors and individual patterns of testosterone

**Figure 2:**
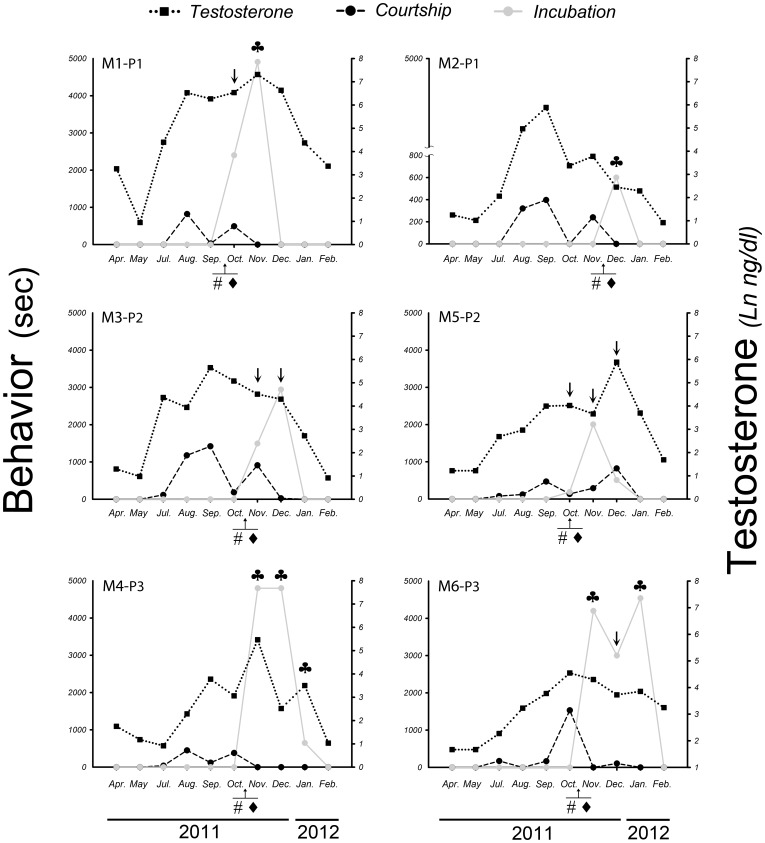
Individual profiles of testosterone and reproductive behaviors in Greater Rhea adult males. Annual changes in testosterone plasma levels of adult males 1 to 6 (black squares-dotted line). The reproductive behaviors: courtship (black circles-dashed line) and incubation (grey circles-grey solid line) were only observed during the reproductive period (August to January). Nest building is indicated with **#**, whereas the first egg detected in each pen is indicated by ♦. Arrows (↓) indicate the presence of two reproductive behaviors (courtship and incubation) on the same day. Incubation behavior for one month or more is indicated with clovers (♣).

All the rhea males showed reproductive behaviors (courtship, nest building, incubation) only during the reproductive period for this species (August to January) (Fig. 2). Our results indicate that, as expected, reproductive behavior of Greater rhea males in captivity coincide with those reported in the wildlife [5], [6], [28]. In all the studied males, testosterone levels remained high throughout the reproductive period (August through January vs. April and May). Four of the 6 males (M 1, 3, 5, 6) showed courtship and incubation behaviors simultaneously (arrows). Males 1, 2, 4, 6 had incubating behavior over a month, or more (clover mark) and these behaviors were always accompanied by high testosterone levels (Fig. 2).

### 17β-Estradiol levels

**Figure 3:**
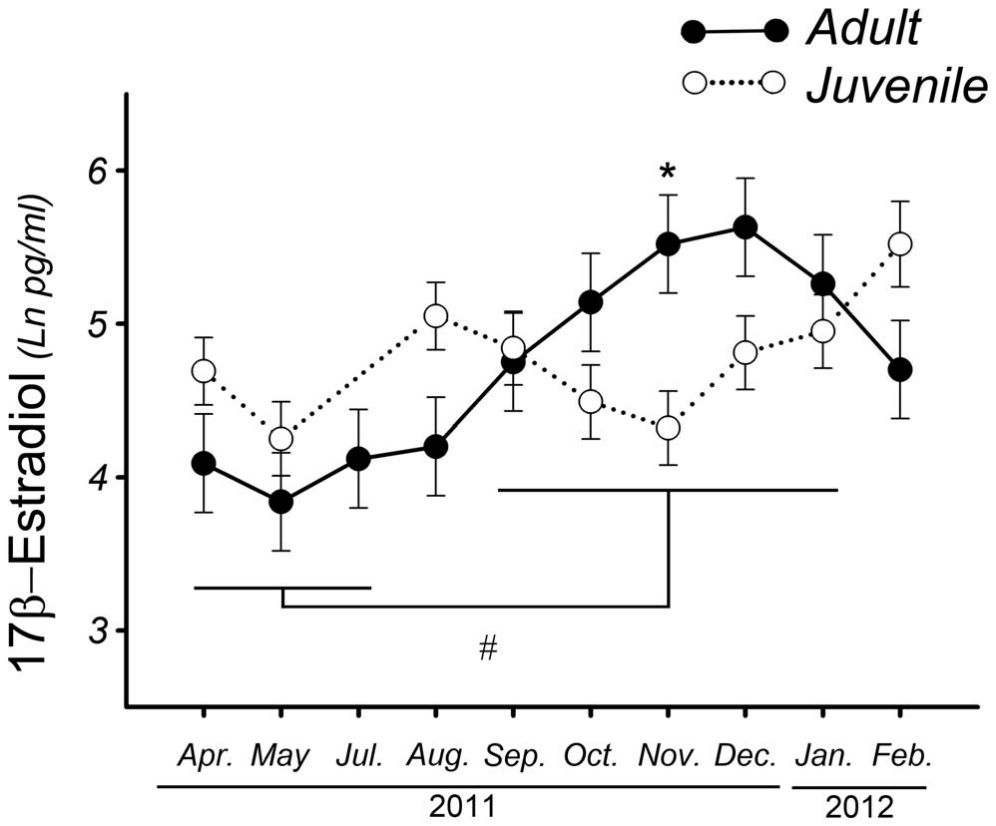
Annual changes in 17β-estradiol plasma levels of Greater Rhea adult females (solid line-filled circle) compared with juvenile rhea females (dashed line-empty circle), showing the differences in November (*). **#** shows differences between April-July and September-January in Greater rhea adult females. Results are expressed as mean ± standard error; adult females n = 11, chick females n = 4; * and # denote *p*<0.01.

Adult female plasma 17β-estradiol levels varied significantly among months (*F*_9, 90_ = 5.98, *p*<0.01, *Mod 4*, Fig. 3). We observed the maximum 17β-estradiol levels in November and December; these levels were significantly higher (*p*<0.01) than those observed in April, May, July and February (non-reproductive period or baseline levels). From July to October, levels were intermediate between the highest and the baseline values.

Juvenile plasma 17β-estradiol levels did not change among months (F_8, 18_ = 2.44, *p*>0.05, *Mod 5*, Fig. 1).

The variations in plasma 17β-estradiol levels among months showed an interaction with breeding status (F_8, 98_ = 3.49, *p*<0.01, *Mod 6*, Fig. 3). The plasma 17β-estradiol level in adults was greater (*p*<0.05) than that of juveniles in November.

The coefficients of variation for nontransformed 17β-estradiol concentration (December, pool n = 15) were 1.76% (intra-assay) and 14.92% (inter-assay).

### Egg laying period

Egg laying was observed throughout the reproductive season and always just after midday, as it occurs in the wild. It began in September (1.3±1.33), increased in October (14.33±6.33) and November (10.66±3.66), and decreased in December and January (3.33±3.33; 5±3.21).

## Discussion

In this paper we report the first findings on the endocrine profile underlying the expression of the mating system of the Greater Rhea. Both male and female adults showed the highest levels of sex hormones all throughout the reproductive season and the lowest ones during the non-reproductive season, in agreement with our hypothesis. These fluctuations reveal the seasonal changes in gonadal function in accordance with their promiscuous mating system. Moreover, and in agreement with our predictions, no significant fluctuations were observed in the sex hormones of juvenile males and females.

An important result of this study is that all Greater Rhea males without exception had high testosterone levels throughout the entire breeding period (September to January), even while they were incubating. According to Martella et al. (1998) [11] and a recent genetic analysis of communal nests in Greater Rheas (Martella et al. unpubl. data), the mating system seems to be promiscuous. This reproductive strategy implies that even while incubating, Greater Rhea males may still be able to copulate with females approaching the nest, thus requiring testosterone levels to remain high.

Another explanation may be that as nests of this species are highly exposed to other conspecific males, reproductive males require high testosterone levels to trigger defensive behavior for protecting either their clutch or their brood [4], [28].

The high testosterone levels during the incubation and rearing periods observed in Greater Rhea males seems to be a common feature in ratites that have communal nests and complex mating systems [29], [30], where males are responsible for the full incubation of eggs, such as the emu [5], [14], or partial incubation, such as the ostrich [2], [5], [13]. The exception within the group is the kiwi (a monogamous species), in which testosterone levels drop when incubation begins [15]. This relationship between high testosterone levels and reproductive behavior (incubation and chick rearing) observed in males of these three species (rhea, emu and ostrich) appears not to conform to the “Challenge Hypothesis” proposed by Winfield in 1990 [3]. Indeed, these species exhibit hormonal profiles similar to those of polygynous species but have monogamous reproductive behaviors (incubation and chick rearing) throughout the breeding period [1], [2], [13], [14]. At variance with the predictions of “The Challenge Hypothesis" as verified in most birds, in ratites there is apparently no trade-off between parental behavior (incubation) and reproductive behavior. High testosterone levels are beneficial to the defense of the nest against conspecific males, courtship behavior, copulation but does not inhibit the incubation behavior. However, the physiological costs of maintaining high testosterone levels for prolonged periods have been widely reported. Among other consequences this hormonal profile causes decreased immune function [31], [32] and decreased muscle mass [33]. Thus, the cost-benefit of maintaining constantly elevated levels of testosterone throughout the breeding period should be further evaluated in *Rhea americana*.

As we predicted, the highest estradiol levels in Greater Rhea females coincided with the period of maximum number of eggs laid. Interestingly, from October to December estradiol levels remained high and steady, but most eggs were laid in October. This phenomenon might be due to the lack of synchronization among the females; they move in groups that are neither stable nor cohesive, they copulate with different males and successively lay eggs in different nests [34], [35] and thus maintain high estradiol levels throughout the entire reproductive season. This observation is in accordance with findings reported for the female ostrich [13] but not for the female kiwi [15] or other non-ratite monogamous birds, whose estradiol level decreases after the first egg is laid [36]–[38]. Similarly to observations reported in the wild, in captivity rhea females egg-laying behavior always occurred just after midday [6], [19].

As expected, the constant levels of testosterone and estradiol in juvenile male and female Greater Rheas, respectively, indicate a lack of seasonal control over gonadal function due to their sexual immaturity [39]–[45]. It was very interesting to find high levels of sex hormones (testosterone and estradiol) in juvenile males and females since we had expected the levels to be similar or lower than the baseline levels of adult (non-reproductive period April-May). One possible source of these hormones is extra gonadal structures such as the adrenal gland or the transformation of steroid precursors in the fat tissue [46], [47]. More studies should be conducted to elucidate the role of high levels of sex hormones in juvenile rheas.

Overall, our findings show seasonal changes in testosterone and estradiol plasma levels in adult Greater Rheas and indicate that contrary to “The Challenge Hypothesis” [3], testosterone might not inhibit parental behavior in males during the breeding period. We also suggest that rhea females might require high levels of estradiol throughout the entire reproductive season in order to be able to copulate with differents males and lay eggs successively in different nests, in accordance with the complex reproductive system exhibited by this species.
